# Characterizing metabolic, behavioural, and bone changes in a novel aging mouse model of sporadic Alzheimer’s disease

**DOI:** 10.1002/alz70861_108387

**Published:** 2025-12-23

**Authors:** Evelin Melekh, Emma L Cook, Amélie AT Marais, Ryan RW Baranowski, Jessica A Mrezar, Shawn M Beaudette, Val A Fajardo, Wendy E Ward, Rebecca EK MacPherson

**Affiliations:** ^1^ Brock University, Department of Health Sciences, St. Catharines, ON Canada; ^2^ Brock University, Department of Kinesiology, St. Catharines, ON Canada; ^3^ Brock University, Centre for Bone and Muscle Health, St. Catharines, ON Canada; ^4^ Brock University, Centre for Neurosciences, St. Catharines, ON Canada

## Abstract

**Background:**

Alzheimer’s disease (AD) is linked to comorbidities such as osteoporosis, sarcopenia, and type II diabetes. Currently, there is a lack of mouse models that mimic sporadic AD (sAD) which accounts for ∼95% of AD cases. A new humanized mouse model (hAb) was developed by substituting 3 amino acids in the amyloid beta sequence to the human counterpart. These mice develop an age‐dependent sAD like pathology. This study characterized age‐related changes in body composition, energy expenditure (EE), movement, bone health, glucose tolerance and insulin sensitivity prior to the development of sAD in the hAb mice.

**Methods:**

Wild type (WT) and hAb mice (*n* =10 per group) were characterized from 4 to 16 months of age. At the age of 4 months, and repeated every two months, body mass, whole body lean and fat mass, and the bone mineral density (BMD) of whole body, tibia, femur, and lumbar vertebrae 1‐5 were measured using dual‐energy X ray absorptiometry (DXA). Glucose and insulin tolerance testing (GTT & ITT) were also performed. EE and movement were measured using metabolic caging, and novel object recognition (NORT) and location (NOLT) testing were performed at age 4 and 16 months.

**Results:**

The hAb mice significantly gained more weight and fat compared to WT throughout aging. They also had significantly lower percentage of lean mass and faster rates of lean mass decline throughout aging compared to the WT mice. EE and movement were also lower in the hAb mice. No genotype effects were seen in whole body and regional BMD or the area under the curve for GTT and ITT. The hAb mice spent less time with the novel object in the NORT and NOLT, however this result was not significant.

**Conclusion:**

The increased fat, and decreased percentage of lean mass along with reduced movement and energy expenditure in the hAb mice may indicate metabolic dysfunction which may precede sAD development. Future steps include increasing the sample size and investigating sex differences between the hAb female and male mice to determine any sex specific changes.

